# Circadian regulation of pulmonary disease: the importance of timing

**DOI:** 10.1042/CS20220061

**Published:** 2023-06-14

**Authors:** Peter S. Cunningham, Callum Jackson, Amlan Chakraborty, Jafar Cain, Hannah J. Durrington, John F. Blaikley

**Affiliations:** 1 Faculty of Biology, Medicine and Health, The University of Manchester, Oxford Road, Manchester M13 9PL, U.K.; 2 School of Mathematics, The University of Manchester, Oxford Road, Manchester M13 9PL, U.K.; 3 Wythenshawe Hospital, Manchester University NHS Foundation Trust (MFT), Southmoor Road, Wythenshawe, Manchester M239LT, U.K.

**Keywords:** asthma, chronic obstructive pulmonary disease, circadian clock, infection, Lung, pulmonary fibrosis

## Abstract

Circadian regulation causes the activity of biological processes to vary over a 24-h cycle. The pathological effects of this variation are predominantly studied using two different approaches: pre-clinical models or observational clinical studies. Both these approaches have provided useful insights into how underlying circadian mechanisms operate and specifically which are regulated by the molecular oscillator, a key time-keeping mechanism in the body. This review compares and contrasts findings from these two approaches in the context of four common respiratory diseases (asthma, chronic obstructive pulmonary disease, pulmonary fibrosis, and respiratory infection). Potential methods used to identify and measure human circadian oscillations are also discussed as these will be useful outcome measures in future interventional human trials that target circadian mechanisms.

## Introduction

Circadian biology regulates time-of-day responses within the respiratory tract. Recent evidence suggests that this could be a key driver of respiratory pathophysiology regulating both inflammatory and fibrotic processes. Some of these findings have been summarised in recent reviews [1] focusing on animal models. For instance, in these models, cytokine expression in response to lipopolysaccharide (LPS), a bacterial endotoxin, is 3-fold higher in the morning compared with administration in the evening [2], resulting in increased neutrophilia and mortality. Based on this and similar studies using other animal models, circadian regulation of respiratory diseases is now an area of active interest. This review will compare the findings from disease models and human studies to highlight both the translational importance of circadian biology and likely relevant mechanistic pathways.

The importance of circadian biology extends beyond the respiratory tract [3]. A recent workshop [4] summarised the evidence linking circadian biology to medicine, especially regarding mental health, metabolic, cardiovascular, gastrointestinal, and rheumatological diseases. These diseases show time-of-day regulation of pathological processes, e.g., inflammation. These circadian responses are dependent on the animal, organ, and stimuli being studied. For instance, in nocturnal animals, a peak response often occurs during the dark contrasting with diurnal animals where the same peak response usually occurs during the day [5]. The exact timing of the peak response is also organ specific, influenced by the relevant organ's underlying circadian oscillation [6]. Finally the stimulus evoking the response is also important, for example the peak responses to bacterial endotoxin (LPS) and bleomycin in the respiratory tract occur almost 12 h apart [2,6] at ZT0 (zeitgeber time 0, lights on) and ZT12 (lights off). Therefore, when comparing studies it is important to consider the diurnal nature of the animal, the organ or cell type and the stimulus as these can often explain what appear at first glance to be discrepant results.

This review will therefore discuss the conserved central mechanism of the peripheral oscillator (clock) alongside common circadian terminology so that each circadian study can be put into a mechanistic context. The circadian regulation of pathophysiological mechanisms for four respiratory diseases (asthma, chronic obstructive pulmonary disease [COPD], pulmonary fibrosis, and infection) will then be discussed comparing findings from both pre-clinical and clinical studies. Finally, the mathematical analysis of circadian rhythms will be discussed since accurate assessment of circadian rhythms will be essential to apply circadian logic in clinical practice.

## Characteristics of a circadian oscillation

**Figure 1:**
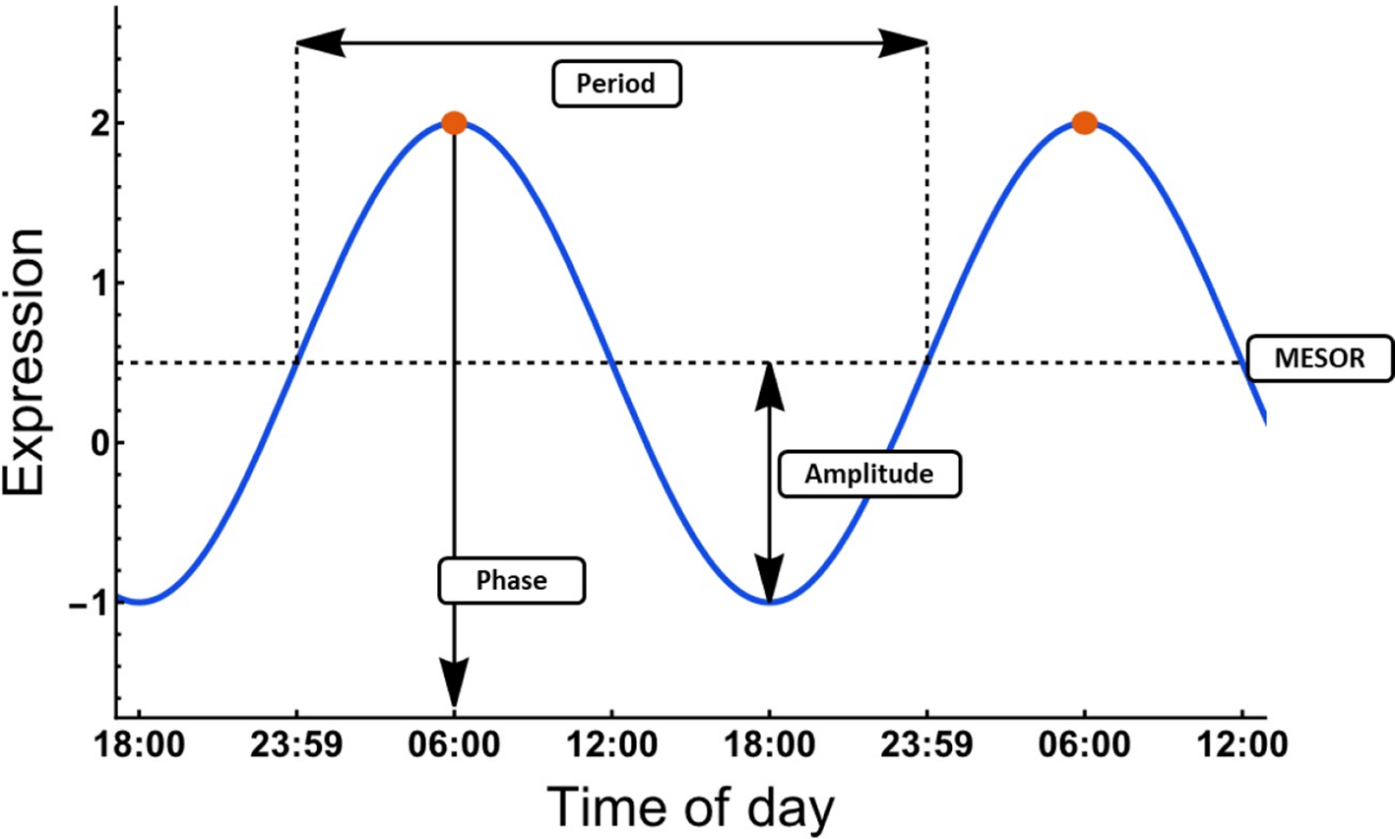
A perfect sinusoid representing a circadian oscillation The four key parameters have been labelled: MESOR is the baseline of the oscillation; period is the time taken for the oscillation to complete one full cycle; amplitude is the distance from the MESOR (baseline) to the trough (equivalently calculated as the distance from the MESOR to the peak); phase is the time of day associated with the peak of the wave. For this example, MESOR = 0.5, period = 24 h, amplitude = 1.5 and phase = 6 am.

The parameters of a circadian oscillation can be described using four parameters known as the MESOR, period, amplitude, and phase. The *MESOR* is an analogue of the mean. The difference between these measures is that the MESOR (Midline Estimating Statistic Of Rhythm) is less affected by the frequency or period of sampling than the mean as it takes into account the oscillation. Therefore, the MESOR provides a baseline around which the rhythm oscillates (Figure 1). Next, the *period* indicates how long it takes the oscillation to complete one full cycle. To calculates the period, measurements can be taken from any two points spaced exactly a cycle apart, e.g., peak-to-peak and trough-to-trough (Figure 1, period is measured from when the oscillation first crosses the MESOR). By definition, the period of a circadian oscillation is approximately 24 h. Lengthening the period means the oscillatory peaks occurs less frequently, and conversely shortening the period causes these peaks to occur more frequently. The *amplitude* of a wave is half of the distance between the oscillation’s peak and trough. It can also be defined as the distance from the MESOR (baseline) to the peak or trough (Figure 1). Lastly, the *phase* of the oscillation is the time of day associated with the peak of the oscillation. Changing the phase of an oscillation alters the time that the peak of the wave occurs but does not alter the shape of the oscillation (Figure 1). Importantly the distribution of phases of rhythmic genes is not evenly spread with transcriptionally ‘busy’ times observed as cells and tissues transition though different states (poised, derepression, activation, transcription, and repression) [7]. Specifically in the lungs, this occurs in the early morning (ZT1-5) in non-human primates [5].

## The transcription-translation feedback loop

**Figure 2:**
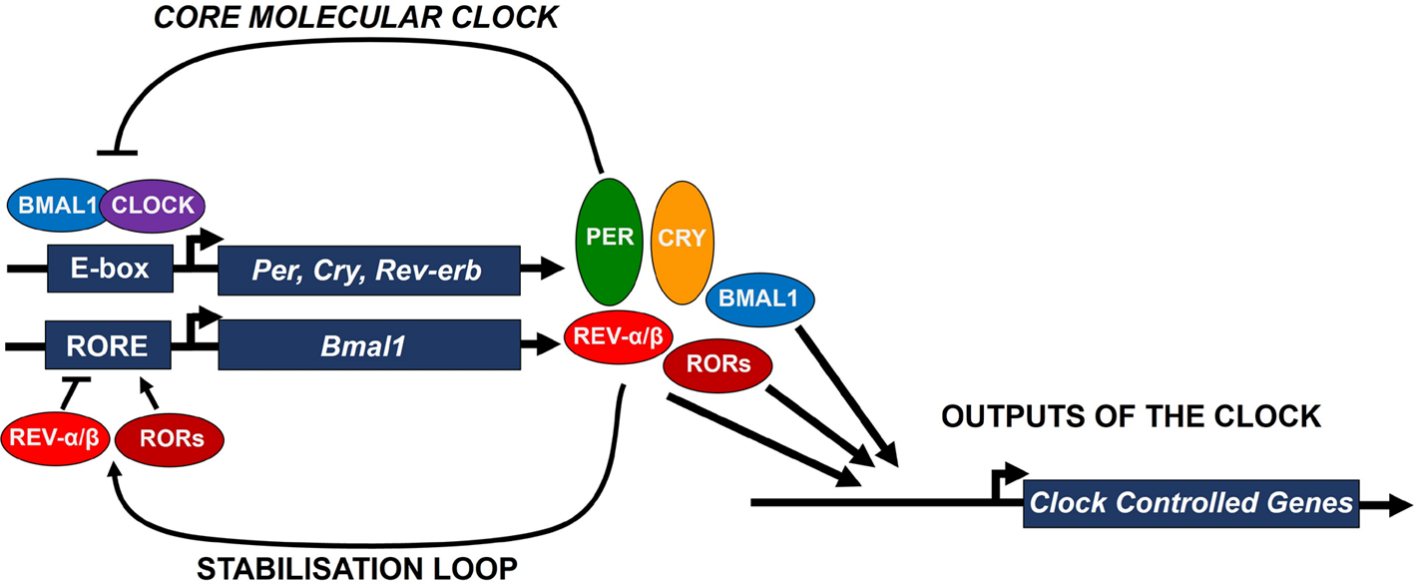
The mechanism of the peripheral oscillator This oscillates with a 24-h period through a transcription translation feedback loop (TTFL). BMAL1/CLOCK heterodimer drives the transcription of PER and CRY. These proteins then inhibit the BMAL1/CLOCK heterodimer creating a negative feedback loop. Over time PER and CRY degrade, partly through the actions of SIRT1, enabling a new cycle to start. REV-ERB inhibits the transcription of BMAL1 which provides a further level of control to the feedback loop. In contrast ROR activates the transcription of BMAL1 antagonising the action of REV-ERB (REV-α = REV-ERBα; REV-β = REV-ERBβ; E-box = enhancer box DNA response element; RORE = RAR-related orphan receptors response element).

The cellular machinery that drives circadian oscillations is present in most cells and is termed an oscillator [7]. This oscillator comprises a transcription-translation feedback loop (TTLF), which regulates the expression of clock genes over a 24-h period. The activators, or positive arm of the clock, consists of BMAL1 (also known as ARNTL) and CLOCK (Figure 2). These transcription factors heterodimerise to form a CLOCK-BMAL1 complex. This complex binds to E-box elements, driving the expression of the repressor, or negative arm of the clock. This repressor arm consists of three PERIOD genes (*PER1, PER2,* and *PER3*) and two CRYPTOCHROME genes (*CRY1* and *CRY2*). Accumulation and translocation of PER and CRY into the nucleus permits interactions with the CLOCK-BMAL1 complex, repressing their own expression creating a negative feedback loop. These proteins are then targeted for degradation (Figure 2) eventually removing this repression, permitting the cycle to restart driven by the activators BMAL1 and CLOCK.

This core molecular clock oscillation can be modified through direct regulation of *BMAL1* expression by REV-ERB and ROR. These two nuclear receptor subfamilies act to stabilise the core clock providing precision and robustness to the molecular timer [8]. REV-ERBs (REV-ERBα and β), whose expression is also E-box mediated, binds to ROR response elements (ROREs) within the *BMAL1* promoter repressing transcription. In contrast, the ROR family of transcriptional activators (RORα, RORβ and RORγ), whose rhythmic expression is mediated via DBP and NFIL3 D-box binding [7] bind to the same consensus ROREs activating transcription.

While this interplay is occurring within the core clock, these transcriptional activators, repressors and co-repressors also regulate a significant number of genes outside the clock (termed ‘clock-controlled genes’) driving the rhythmic expression of many biological pathways (Figure 2). Downstream regulation by clock-controlled genes is highly tissue- and context-specific. In the lung their effects have been shown to regulate both physiology (normal lung function) and pathophysiology (e.g., inflammatory responses, DNA damage/repair, and oxidative stress responses) [9]. In recent years additional levels of detail have been described for the molecular clock, including post-transcription regulation and protein modification, such as oxidation and phosphorylation, and examples of circadian oscillations without classic TTFLs [10]. This highlights the complexity of the circadian mechanism and shows some of the challenges when trying to elucidate the effects of both systemic and local circadian disruption.

## Models of circadian dysfunction

Circadian research in respiratory biology often uses animal models of circadian dysfunction to investigate circadian control. To interpret these studies alongside observational clinical studies, it is important to understand the advantages and limitations of the common animal models used. Due to the existence of several redundant components in the molecular oscillator, it is only possible to cease circadian oscillations by targeting specific checkpoints. A frequently used checkpoint is targeting BMAL1 by knocking it out. In this model, loss of BMAL1 causes cessation of the TTFL and reduced REV-ERBα expression [11]. As with other knockout models, the observed effects of BMAL1 could be attributed to the regulation of this protein rather than to circadian oscillations. Therefore, findings from this model will often be combined with additional circadian animal models to demonstrate circadian control. These additional models will either target downstream circadian pathways, e.g., REV-ERBα or use another genetic modulation to stop circadian oscillations. Two other mouse models can accomplish this, either a CRY1/2 double knockout [12] or a dominant negative mutation of the CLOCK protein, termed Clock∆19 [13]. Beyond targeting cessation of the clock, other clock knockout models can be applied to investigate the role of specific clock protein isoforms. For example, specific deletion of *Per2*, modelling circadian rhythm disruption, accelerated lung tumorigenesis [14] and deletion of *Per3* identified period and phase shifts in the lung that were not observed in the suprachiasmatic nucleus, suggesting a role for PER3 in peripheral clock regulation [15]. These examples highlight the myriad tools currently available to circadian researchers.

Within the lung, numerous cell types have been identified that confer cell-specific circadian regulation, including epithelial cells [16], immune cells [17–19], and fibroblasts [19]. To determine the responsible cell type a Cre-lox recombination system can be used, knocking out proteins in specific cell types. This system can also be used to knock out BMAL1 following birth, delineating developmental effects from other phenotypes. Several different circadian modified genetic models have been studied in the context of pulmonary disease and these will be further described later in this review.

Targeting circadian proteins is impossible in humans and therefore environmental manipulation of the clock inputs (sleep, light, food) has been used to try and elucidate downstream targets. This environmental manipulation can be mimicked in animals however the effects of the stimuli vary between species. One common protocol is termed the jetlag protocol [20] where the phase of the clock is advanced between 4 and 8 h every week. This protocol may partially simulate either shift work or travel between different time zones. With the advent of modern lighting however this phase advance is also equivalent to social jetlag where the normal rhythmicity in the working week is disrupted at weekends by either staying up late at night or having a lie in [21]. Another form of environmental manipulation is constant light or constant darkness [22] where the animals, often mice, are exposed to an environment without any timed lighting cue. Both of these stimuli can dampen circadian oscillations, however recently constant darkness has been linked to an exaggerated inflammatory response which may be independent of any circadian regulation [23].

Therefore, there is no ideal stimulus or model to study the loss or gain circadian rhythmicity. Currently investigators rely on conserved findings across different models of circadian disruption to confirm that a pathophysiological process is under genuine circadian control.

## Circadian regulation of asthma

**Figure 3:**
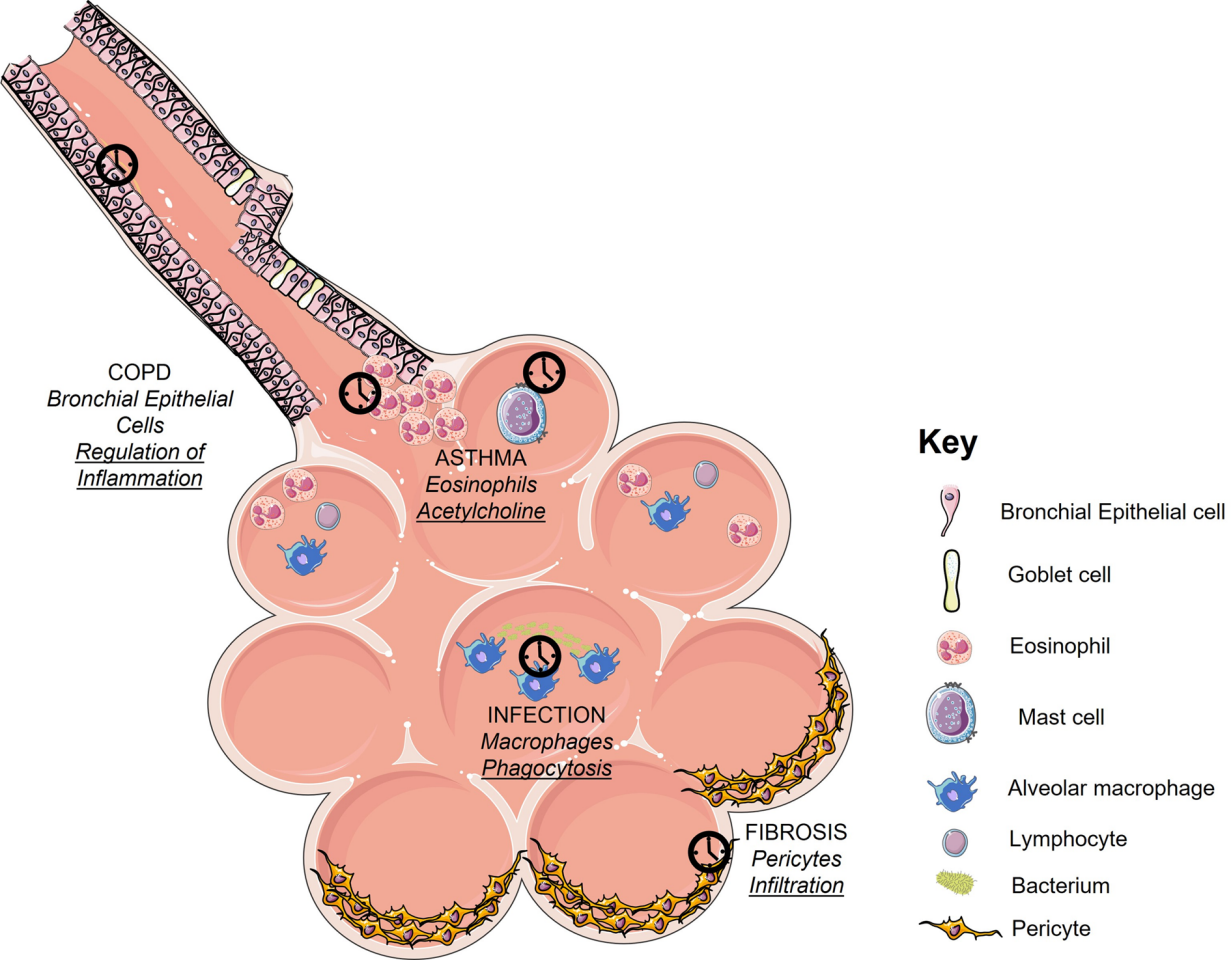
Cell types involved in pulmonary circadian pathology In the lung, circadian effects can be mediated through different cell types, dependent upon the underlying disease. For instance, the peripheral oscillator regulates eosinophil chemotaxis a key determinant of eosinophilia, a marker of severe asthma. Inflammatory responses to cigarette smoke, a key aetiological agent in COPD, is under circadian control in the club cell. Fibroblast/ myofibroblast differentiation, partly responsible for the deposition of collagen in pulmonary fibrosis, is also under circadian control. Phagocytosis of bacteria by macrophages is also regulated by the peripheral oscillator, which is important in pneumonia. Therefore, circadian regulation of pulmonary pathophysiology is mediated through several different cell types and mechanisms.

**Figure 4:**
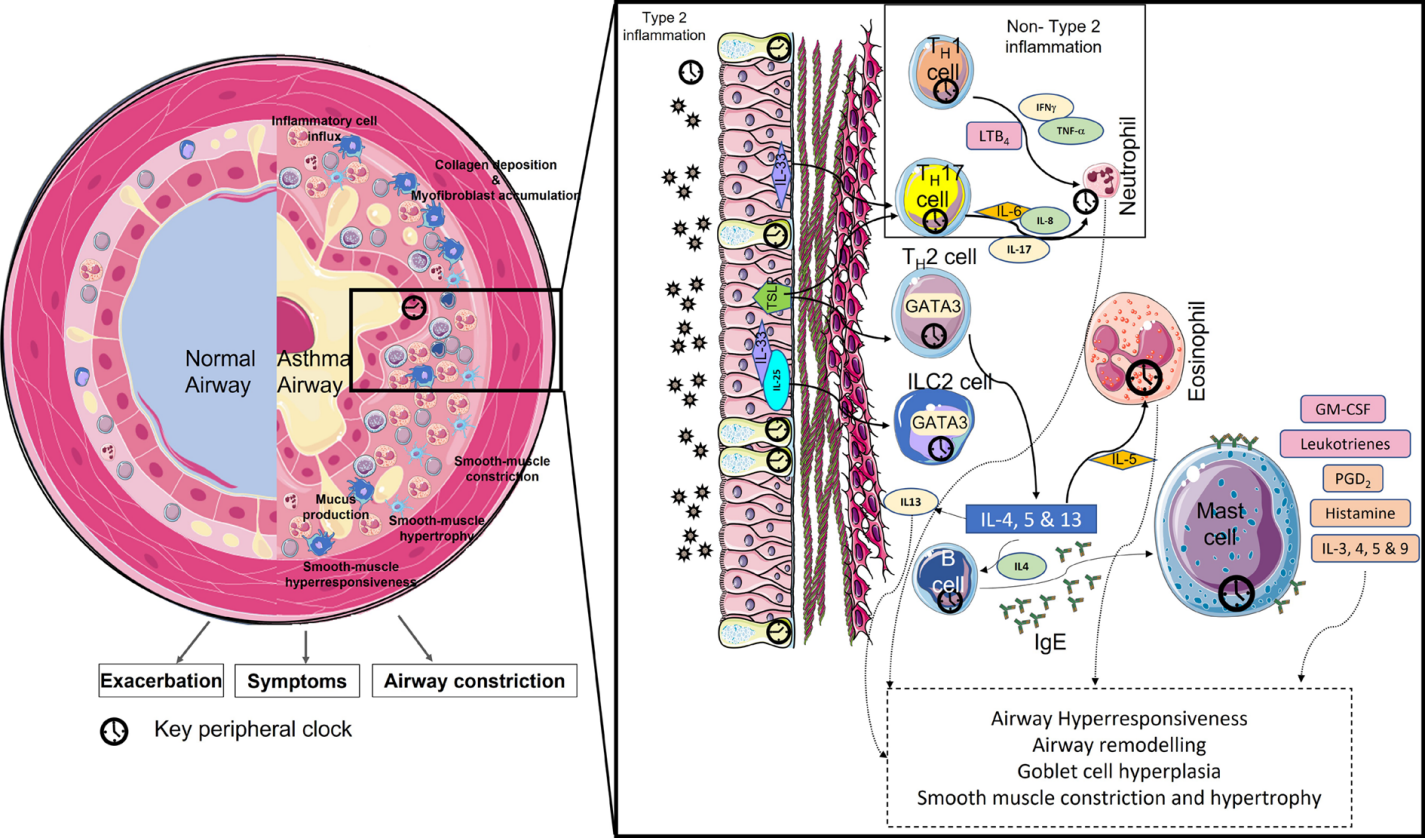
Circadian regulation of the pathophysiology of asthma Asthma is characterised by airway constriction and inflammatory cell influx leading to airway hyperresponsiveness and airway remodelling associated with smooth muscle hypertrophy, goblet cell metaplasia, and accumulation of myofibroblasts and collagen. Type 2 inflammation is initiated by an adaptive immune response due to exposure to allergen, stimulating T-Helper 2 (TH2) cells and Innate Lymphoid group 2 (ILC2) cells to secrete interleukins (IL)- 4, 5, and 13. IL-4 stimulates the production of allergen specific IgE antibodies from B cells causing mast cell activation and degranulation leading to secretion of histamine, IL-3, 4, 5, 9, and prostaglandin D2 (PGD2). IL-13 aggravates AHR, stimulates goblet cells to produce mucus and airway epithelial cells to produce cytokines/chemokines for eosinophil recruitment. IL-5 secretion leads to eosinophil trafficking which is regulated by the clock and demonstrates a time-of-day effect. Non-type 2 inflammation also occurs in asthma, through activation of the innate immune response resulting in T-Helper 1 (TH1) and T-Helper 17 (TH17) cells leading to neutrophil recruitment. Figure adapted based on Durrington et al., 2018 [44], Lloyd et al., 2001 [35], Gibbs et al., 2009 [16], and Israel et al., 2017 [143].

The Global Initiative for Asthma (GINA) defines asthma as a heterogeneous disease characterised by chronic inflammation resulting in airway hyperresponsiveness (AHR) with symptoms such as wheeze, shortness of breath and chest tightness varying over time [24]. It is now recognised that there are many distinct phenotypes of asthma. With the development of therapeutic monoclonal antibodies directed against various Type 2 inflammatory proteins, it is now clinically useful to classify asthma as either Th2 high or low. The most prevalent type is allergic asthma, or asthma characterised by eosinophilia and strong IgE mediated responses [25], also known as type 2 (T2 high) asthma. Asthma, like other atopic diseases can be triggered by a range of allergens, most commonly house dust mite allergen [25,26] and others such as pollution, pollen, and pet dander. We and others have shown that the timing of allergic challenge can determine the magnitude of inflammatory response and pathobiology of asthma [27] (Figures 3 and 4). Specific examples of this circadian regulation include the diurnal variation in eosinophils [28], the circadian regulation of the expression of IL-6 and IL-13 in mast cells expressing high affinity IgE receptor (FceRI) [29] and the circadian regulation of basophil degranulation in response to IgE ligation in asthma [30].

### Asthma models

While mice do not naturally develop asthma, *in vivo* models have been found to successfully replicate many of the inflammatory changes which are characteristic of the human disease, namely airway hyperresponsiveness (AHR), eosinophilia, and Th2 cell responses [31–33]. Two animal models are often used; Ovalbumin or the House Dust mite model. The Ovalbumin model involves a period of sensitisation followed by a challenge phase [31,34], producing mild levels of AHR [35], tissue remodelling (including goblet cell hyperplasia) [31,36] and increased eosinophil counts in bronchoalveolar lavage fluid [29]. Models using house dust mite (HDM) allergens often produce a more robust inflammatory response compared to the Ovalbumin model and seem to more closely mimic human disease thanks to the use of an allergen which is frequently implicated in human asthma [26]. HDM models involve repeated intranasal exposure of an animal to the *Derp1* allergen. *Derp1* allergens have intrinsic cysteine-protease characteristics resulting in a more profound AHR [30] and help to prevent the emergence of tolerance to the allergen [32].

Using the HDM model of allergic airway disease and an *ex vivo* precision cut lung slice (PCLS) model, Durrington and colleagues identified a pathway linking the core molecular clock, through REV-ERBα to airway reactivity, smooth muscle tone, and airway narrowing. They found that time-of-day effects in AHR following allergen challenge were ablated in REV-ERBα-deficient mice, yet, allergic inflammation increased overall. Rhythmic expression of key muscarinic receptor sub-classes, mediating cholinergic smooth-muscle responses were also lost in REV-ERBα-deficient mice [37]. Furthermore, loss of *REV-ERBa* expression in the lung appears to be a feature of human asthma [27] and the HDM mouse model [37].

Mice lacking BMAL1 in myeloid cells (*BMAL1-LysM^−/−^*) were used to determine the role of BMAL1 in allergic asthma. Using the ovalbumin model of allergic asthma, *BMAL1-LysM^−/−^* mice demonstrated markedly increased asthma features (increased lung inflammation, eosinophils as well as increased IL-5 levels in the lung and serum). Subsequent *in vitro* studies demonstrated that macrophages from *BMAL1-LysM^−/−^* mice had increased proinflammatory chemokine expression following LPS stimulation or mannose receptor expression following IL-4 stimulation [38]. Ehlers and colleagues used a different asthma model to investigate the role of BMAL1 in asthma. Deletion of the core clock protein BMAL1 or environmental disruption of circadian function by jetlag exacerbated viral bronchiolitis caused by Sendai virus (SeV) or influenza A virus (IAV) in mice. Importantly, *BMAL1^−/−^* mice developed much more extensive asthma-like airway changes post-infection, including mucus production and increased airway resistance suggesting a role for BMAL1 in the development of asthmatic airway disease via the regulation of lung antiviral responses to common viral triggers of asthma [27] (discussed in Section 7).

A useful alternative which mimics most of the features of the *in vivo* models utilises bronchial epithelial cell culture at the air–liquid interface. Air–liquid interface models of airway epithelial cells are considered a gold standard for studying the primary epithelium and the transcriptional regulation of an intact tissue. Air–liquid interface cultures of cells undergo extensive differentiation at the mucociliary level which is a true representation of an actual airway *in vivo*. For this purpose, they have been extensively used *in vitro* for understanding mechanisms involving epithelial damage in asthma. Air–liquid interface cultures are useful in testing drug formulations for inhalation delivery in the form of aerosol particles or dry powder on to the surface of the ciliated epithelial layer, mimicking the deposition of powders on to the lung surface *in vivo* [39,40]. Moreover, air–liquid interface models of airway epithelial cells serve as a novel tool to understand the regulation of clock genes in the bronchial epithelium. Zhang and colleagues showed the importance of CCSP expressing bronchiolar epithelial cells in controlling pulmonary responses to influenza viral infection using an air-liquid interface model [41].

### Human studies in Asthma

**Figure 5:**
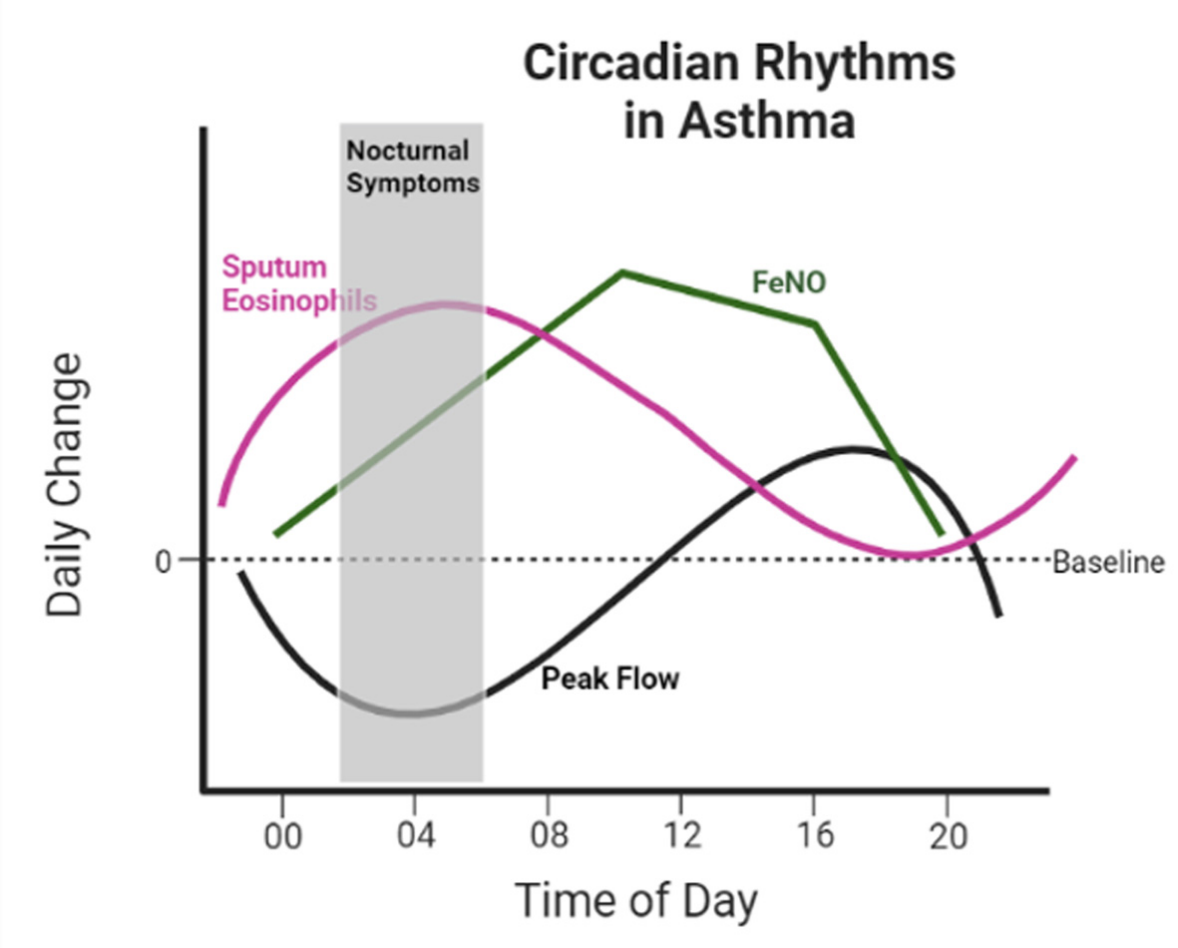
Circadian rhythms in clinical asthma Symptoms of asthma worsen during the night, peaking around 04:00 [45]. This is mirrored by a corresponding decrease in lung function (shown here as peak flow) and increase in sputum and blood eosinophils both at 04:00 [44]. Lung function is highest at 16:00 [44,45]. Fractional exhaled nitric oxide (FeNO) also shows a diurnal variation, peaking at 10:00 [47].

Asthma is a rhythmic inflammatory disease of the airway, characterised by marked diurnal symptoms. Wheeze, cough and breathlessness worsen overnight/early morning [42]. Mortality associated with asthma has a strong time of day influence, peaking between midnight and 08:00 hours [43]. The AHR associated with asthma demonstrates diurnal variation with an early morning peak (04:00 hours) which influences the pathophysiology of the disease. Airway narrowing, measured clinically by spirometry (Forced Expiratory Volume in 1 second (FEV_1_)) or Peak Expiratory Flow (PEF) fluctuate over the day in healthy individuals with a nadir at 04:00 hours; however, this is greatly magnified in asthma patients [43,44]. Recently, airway calibre in asthma has been shown to be regulated by the circadian system [45]. Airway inflammation in asthma also seems to vary by time of day. Sputum eosinophils peak around 04:00 in asthma [44] and fractional exhaled nitric oxide (FeNO), a breath biomarker of eosinophilic inflammation used in the clinic, peaks around 10:00 [46] (Figure 5). Durrington and colleagues have also shown that fluctuations in sputum eosinophil counts occur within the clinical working day in severe asthma and could influence treatment decisions [44]. Furthermore, there is diurnal variation in novel asthma biomarkers such as the extra-cellular matrix protein Periostin in serum [46] and breath volatile organic compounds (VOCs) [47].

Epidemiological studies by Maidstone and colleagues have demonstrated disturbances in the biological clock disrupting internal circadian time affecting the development of asthma, with night-shift workers susceptible to an increased risk of asthma and metabolic disorders [48]. Lastly, the identification of polymorphisms in clock genes (e.g. TIMELESS) have also been associated with increased risk of asthma in children [49].

Chronotherapy is the timing of treatment to coincide with disease rhythmicity. Asthma is well suited to chronotherapy. Inhaled corticosteroids (ICS) are by far the most effective drugs used in the treatment of asthma [50]. ICS suppress inflammation within asthmatic airways by inhibiting the recruitment of inflammatory cells into the airway. This suppression of mucosal inflammation is relatively rapid with a significant reduction in eosinophils detectable within 6 h, which is associated with reduced AHR [51]. To date there have been several chronotherapeutic studies involving ICS in asthma. These have shown once daily afternoon dosing with ICS (mometasone furoate [52]) appears more effective than morning dosing. Furthermore, administering the total daily dose of ICS (inhaled triamcinolone [53,54]) once a day in the afternoon is as effective as giving divided doses throughout the day. If these findings hold true in the real world setting, it would imply that current dosing schedules could expose patients to excess risks by dosing at times of day which are not effective.

## Circadian biology in COPD

COPD causes narrowing of the airways (referred to as chronic bronchitis) and destruction of the lung parenchyma (referred to as emphysema). Typically, this condition is caused by a cigarette smoke inducing an inflammatory response which causes pulmonary damage. Although the airway narrowing in COPD is less reversible than asthma, inhalers are still used for bronchodilation (β2 agonists, anticholinergic compounds) and repression of inflammation (corticosteroids).

### COPD models

To model COPD, mice can be exposed to cigarette smoke. Studies of core oscillator components in this model have revealed that cigarette smoke regulates clock component expression at both transcript and protein levels. Cigarette smoke represses the expression of BMAL1, REV-ERBα, and PER2 [55,56] in contrast the expression of RORα [56] is increased. This altered expression of clock transcripts has the potential to affect the oscillatory dynamics of the system. This seems to be the case since after exposure to cigarette smoke, the amplitude of PER2’s oscillation is dampened [56] compared with mice who have not been exposed to cigarette smoke whilst other facets of its circadian oscillation (phase and period) were unaffected.

The consequences of circadian control have been investigated using various genetic knockout models resulting in cessation of circadian oscillations or disruption of downstream pathways. This is exemplified by stopping circadian oscillations, through knocking out BMAL1 in club cells [56] (Figure 3). In this model, the pro-inflammatory effects of cigarette smoke were increased, suggesting circadian modulation of the inflammatory pathway in COPD. To further support a circadian mechanism downstream regulators of the circadian response have also been examined. In this model, there were apparent discrepant findings between study groups [57–59]; however, this could be potentially explained by the length of cigarette smoke exposure. If REV-ERBα knockout mice were exposed to cigarette smoke just once, then they did not have a differential response compared with littermate controls. If cigarette smoke exposure was continued on a daily basis for 10 days, one group [57] reported that knocking out REV-ERBα did not affect the inflammatory response, whilst another study found that it increased the expression of inflammatory mediators (IL-6, KC, and BAL neutrophilia) [59]. The increased inflammatory response to cigarette smoke was also reported with longer, sub-chronic (30 days) exposure [59]. In addition to modulating inflammation, the BMAL1/REV-ERBα axis has also been reported to alter epithelial mesenchymal transition [58], a key process in both COPD [60] and pulmonary fibrosis. Therefore it is likely that the BMAL1/REV-ERBα axis regulates chronic but not acute cigarette smoke exposure. Interestingly, two studies [58,59] have reported beneficial effects of targeting REVERBα with synthetic ligands, suggesting that this mechanism could be targeted therapeutically.

### Human studies in COPD

Studies in COPD patients have confirmed the finding that circadian transcripts are differentially regulated in COPD animal models. Specifically, the clock genes *BMAL1, PER2,* and *REV-ERBα* [61] are reduced concordant with findings in animal models. In addition, the expression of *CRY1* was reduced and *PER1* transcript expression was increased. The mechanism for these changes has still yet to be determined. The effects of hypoxia could be one explanation, as this shifts phase of circadian oscillations resulting in altered clock gene expression if only one timepoint is measured [62]. Another possible explanation is the repression of SIRT1 [63–65] by cigarette smoke. SIRT1 is a deacetylase, promoting the degradation of PER2 which is repressed in COPD. The repression of PER2 in turn could modify TTFL oscillations due to changing the negative feedback loop [11]. Finally, inflammation directly regulates clock gene expression, for instance repressing *REV-ERBα* [57] which is also repressed in COPD.

The relationship between polymorphisms for circadian clock genes have also been examined in COPD. In a small study (450 cases), no link was discovered between circadian polymorphisms and COPD [66]; however, this does not preclude a link being discovered in larger studies. Investigators have also investigated the role of environmental circadian factors in the development of COPD. This has revealed that shift workers, predicted to experience circadian strain, have an increased risk of developing COPD [48] compared with non-shift workers.

The clinical impact of these finding is currently being explored. Intuitively, the suppression of circadian oscillations and reduction in circadian transcript expression would be expected to result in suppression of circadian control. With regards to symptoms however this is not the case, since COPD symptoms are worse in the morning compared with the evening [67–69]. This diurnal oscillation is consistent with that found in asthma, suggesting that other mechanisms (e.g. neuronal or positional) could be responsible. Alternatively, circadian disruption could cause previously hidden diurnal regulatory pathways to emerge. The role of circadian biology in driving this oscillation is likely to be an important area of investigation due to the association between the diurnal oscillation of symptoms and patient morbidity [68,70].

## Circadian biology in pulmonary fibrosis

Pulmonary fibrosis is an irreversible chronic lung condition with many different subtypes. Idiopathic pulmonary fibrosis (IPF) is the most prevalent subtype and therefore circadian research has mainly focused on this subtype. The incidence of IPF increases with age, making it 100-fold higher in those over 75 compared with those under 35 years [71]. Circadian biology could be one explanation for this finding since the amplitude of circadian oscillation dampens with age [72] potentially driving pathophysiology in ageing diseases.

### Pulmonary fibrosis models

There are no specific IPF models, therefore animal models investigate the development of pulmonary fibrosis regardless of the cause. Various models exist [73] however the bleomycin model is the commonest type studied. In this model, an inflammatory response is observed around day 7, followed by a fibrotic response occurring 21–28 days [73] after bleomycin administration.

Circadian biology regulates the initial inflammatory response to bleomycin. The magnitude of this response is significantly higher if bleomycin is given at ZT12 (evening) [6] compared with ZT0. This contrasts with the peak inflammatory response to bacterial endotoxin [2] which occurs at the opposite time-of-day (ZT0). The time-of-day response to bleomycin is under direct control of the peripheral oscillator, since the differential response is lost in mice who do not have a functional peripheral oscillator (Clock∆19) [6]. The pulmonary fibrotic response is also increased in these mice, suggesting that the fibrotic as well as inflammatory responses are under partial circadian control [6]. Supporting this hypothesis is the observation that the pulmonary fibrotic response occurs spontaneously with age in BMAL1 knockout mice [74], mirroring the disease phenotype. These knockout mice also do not have a functional oscillator. This putative circadian regulation is likely explained by several interconnecting mechanisms. For instance, circadian factors alter the redox response through modulation of the nuclear factor, NRF2, which regulates the cell’s resistance to oxidative stress [6]. BMAL1 can exert its profibrotic effects through two different pathways. First, knockout of BMAL1 reduces REV-ERBα expression which in turn increases fibroblast to myofibroblast differentiation by regulating the expression of the transcription factor TBPL1 [75,76] (Figure 3). The second pathway is regulation of epithelial mesenchymal transition [58]. Finally, collagen secretion appears to be under circadian control [77]. This will directly regulate secretion of Collagen-1, a key extra-cellular matrix protein in pulmonary fibrosis.

It appears that the dynamics of the peripheral oscillator are also altered in pulmonary fibrosis. Initially it was thought that circadian oscillations in pulmonary fibrosis were dampened in a manner similar to those described in COPD, due to the effect of TGFβ [78] or the presence of a stiff extracellular matrix [79]. In contrast with this prediction, it appears that the amplitude of PER2 oscillations is increased *in vivo* [75]. These apparent contradictory findings could be explained by the increased presence of fibroblasts in pulmonary fibrosis. The amplitude of the peripheral oscillator is increased in fibroblasts compared with alveolar cells [16] and when the peripheral oscillator is removed in fibroblasts the augmented circadian oscillations are no longer observed [75]. An alternative explanation would be the synchronising effects of the pro-fibrotic cytokine TGFβ, since the increased synchrony between different cellular oscillators would augment circadian amplitude in a population of cells [80].

### Human studies in Pulmonary Fibrosis

The expression of clock transcripts are altered in pulmonary fibrosis. Specifically, *PER1/2* expression is repressed and the expression of both *REV-ERBα* and *ARNTL1* (*BMAL1*) are increased [75]. The aetiology of IPF is poorly described; however the aetiology of adult respiratory distress syndrome (ARDS), where pulmonary fibrosis occurs in response to critical care admission has been linked to time-of-day. Patients are more likely to develop ARDS if they receive a lung transplant or are admitted to critical care following an infection in the morning [81,82]. This would be concordant with the findings in the mice bleomycin model, due to the nocturnal nature of mice. Therefore, the timing of exposure to pro-fibrotic agents could be important in the pathogenesis of pulmonary fibrosis. This is supported by epidemiological studies showing an association between factors which increase circadian strain (shift workers, evening chronotype and sleep length) and an increased risk of developing the disease [75,83].

Circadian regulation is of potential therapeutic importance due to the ability to target circadian mechanisms using chemical compounds. For instance, we have shown that knockout of REV-ERBα increases myofibroblast differentiation and that targeting REV-ERBα with ligands reverses myofibroblast differentiation in cell culture and the secretion of collagen from precision cut lung slices [75].

## Circadian biology of respiratory infection

Respiratory infection is a common cause of death worldwide, therefore its circadian regulation could have significant impact on prevalence and mortality rates. This is exemplified by the COVID-19 pandemic where shift work increased the likelihood of being hospitalised by 4-fold [84]. Most of the work concerning circadian regulation of respiratory infection has focused on bacterial or viral infections. These have distinct pathogenic mechanisms, therefore the animal models are discussed separately but translational findings are discussed together.

### Respiratory bacterial infection models

It has been known since 1969 that there is a time-of-day effect for pneumonia since mice inoculated with *S. pneumoniae* at 4 am had worse outcomes compared with mice inoculated at other timepoints [85]. This time-of-day phenomenon persists after controlling for stimuli which could potentially explain the phenomenon, e.g., light or glucocorticoids [86]. This observation combined with the fact that phagocytosis is under control of the clock protein timeless [87] in drosophila, suggested that the molecular oscillator could partially regulate phagocytosis in mammals. Stopping the peripheral oscillator in the club cell had no effect on bacterial load, despite increasing pulmonary neutrophilia [2]. In contrast, targeting the macrophage by knocking out BMAL1 using two different cre drivers (LYSM and CX3CR1) [88] abolished the time-of-day phenotype *protecting* mice from infection and reducing inflammation (Figure 3). The phagocytic behaviour of macrophages was therefore characterised, revealing that knocking out BMAL1 increased phagocytosis by altering the actin cytoskeleton to prime macrophages. Since circadian control has been reported to occur for other bacteria [89,90] these circadian effects are likely to extend to other types of pneumonia, including nosocomial pneumonia.

### Respiratory viral infection models

It has been known for several years that respiratory viral infections could also be under circadian control. In a similar manner to respiratory bacterial infections the timing of inoculation with influenza or parainfluenza alters mortality or viral load. Mice infected with influenza have increased mortality if inoculated at ZT11 compared with ZT23 [91] and for parainfluenza infection mice lost more weight when infected at ZT18 compared with other time points [27]. This time-of-day effect on outcome is likely to be mediated via the molecular oscillator as abolishing circadian rhythmicity by knocking out BMAL1 increased viral protein expression [92], markers of infection [27,93] and pulmonary damage [27,91,93]. The regulation of circadian viral responses appear to be partially regulated by alveolar type 2 cells [93] as opposed to macrophages which regulate the bacterial response. The involvement of different cell types could explain why knockout of BMAL1 exacerbates viral pathogenesis [92] but is protective in bacterial pneumonia [88].

### Human studies in respiratory infection

It is difficult to determine the time of inoculation in humans making it challenging to replicate animal studies. Instead studies have focused on whether complications of pneumonia e.g. ARDS oscillate in a time-of-day variation [82 (please insert hyperlink to reference 82)]. As discussed previously this association has been found, but it could also be explained by factors other than the circadian clock (e.g. staffing).

Risk factors for circadian strain appear to increase the risk of pneumonia. Two studies have reported that short sleep duration increases the risk of respiratory infection [94,95], in contrast a third reported no association [31]. Three independent studies [84,96,97] have suggested that shift work is associated with an increase in the risk of hospitalisation following SARS-CoV2 infection. All these studies did use the same dataset, however a fourth study using a different dataset reported the same phenomenon [98]. Therefore, circadian factors are important in regulating the pathogenesis of respiratory infection.

## Approaches to measuring circadian oscillation

A key challenge of circadian biology is to identify human circadian oscillations in a robust manner permitting associations with the molecular oscillator to be determined. In the lung this is challenging due to the current requirement for repeated sampling. Therefore, most studies use systemic measures to estimate circadian rhythms, however since circadian clock oscillations can differ between organs these at best can only be approximations of what is happening in the lung.

### Indirect biochemical markers

Four main circadian outputs have been used to infer a patient’s circadian rhythm: melatonin, cortisol, core body temperature, and rest–activity cycles [99–101]. Non-circadian stimuli however can influence their measurement, e.g., melatonin is affected by light [102,103] or cortisol is influenced by stress [104]. Therefore, these measures can only approximate what is happening with the molecular oscillator, rather than provide an accurate measurement.

### Physiological outputs

Due to the advances in wearable technology, activity tracker devices can be used to measure heart rate and activity levels. Such devices record measures at a very high sample frequency (every 5 s) making the mathematics easier to determine whether a circadian oscillation exists. Studies have therefore used heart rate data, following adjustment for activity levels and sleep–wake patterns to develop algorithms to estimate circadian rhythms [105,106]. One major advantage of this approach is that data can be accessed and downloaded remotely, permitting circadian rhythmicity to be measured in participants across relatively long time periods.

One of the most common methods to estimate circadian parameters in humans is through accelerometry/actigraphy. This method has multiple advantages, such as being non-invasive, having a high frequency of sampling and being relatively cheap to perform [107]. High-frequency sampling provides a wealth of data permitting analysis by several mathematical methods. Such analysis can reveal highly specific aspects of the rest–activity cycle, e.g., sleep duration [108], sleep regularity index [109], composite phase deviation [110], and intradaily variability [111]. Reviews have highlighted that the sleep regularity index is linked to pathophysiology [112]; however whether this is linked to respiratory diseases has yet to be widely explored.

Another option is to measure temperature. For core body temperature, measurements can be cumbersome, requiring either rectal sensors or thermistor pills that can be swallowed. Skin temperature, however, is more easily measured and has been evaluated to be a useful method of inferring central circadian rhythm [113]. Analogous to how light can confound the results of melatonin, body temperature is highly influenced by sleep. A participant’s sleep–wake cycle is not only driven by circadian rhythms but also sleep debt. This second process therefore can confound any measurement of the circadian cycle.

### Direct measures of circadian clock function

The expression of core clock transcripts which make up the transcription-translation feedback loop can be directly measured. Since the circadian state varies over time it is necessary to measure the circadian oscillation over at least 24, if not 48 h. Therefore, multiple sequential samples need to be collected over this time period. The frequency and period of sampling is limited by adverse effects which change depending on the sampling site. The most common sampling site is blood; however, other sites (buccal swabs and hair follicles) have also been sampled [114,115].

### Algorithms for quantifying circadian rhythms

Multiple mathematical approaches have been developed to measure circadian oscillations. A complete review of each approach is beyond the scope of this review but has been reviewed elsewhere [116–120]. We, therefore, discuss the most common approaches that can be used to assess datasets where only a few samples have been collected.

One of the most popular methods for analysing circadian data is cosinor (and its extensions) [121]. Cosinor allows for efficient quantification of all the parameters of an oscillation. That is, cosinor will simultaneously estimate the MESOR, amplitude, period, and phase (Figure 1). This is a benefit when investigating circadian disruption as quantifying these parameters offers greater insight into the type of disruption occurring. Further, since the foundation of the algorithm is fitting a cosine curve, it is possible to plot both the data and the estimated circadian fit so the researcher can observe how well the cosinor algorithm fits the data. Cosinor is a flexible framework that is not affected by missing data or unevenly sampled data points facilitating the analysis of a wider range of data sets. However, cosinor can be influenced by outliers [122]. This can lead to poor estimates that are difficult to detect. Moreover, cosinor will always produce an estimate for each parameter, even in situations where the data are completely non-rhythmic. This is an issue when analysing critically ill patients as their condition can completely dampen their circadian rhythm but cosinor will not reveal this.

Techniques that have recently been gaining popularity are Bayesian approaches such as Gaussian Process Regression [123,124]. The typical advantage of a Bayesian approach is better uncertainty estimation [125]. Rather than estimating a single number for the different circadian parameters (e.g., the period), Bayesian techniques will suggest a range of values that are most plausible based on the data. This allows the researcher to determine if the period is significantly different between groups or determine if there is any oscillation at all (based on the size of the amplitude). Moreover, Bayesian methods do not assume that the data must fit an exact shape (e.g., a cosine wave), meaning they better model non-sinusoidal data. Such techniques are typically computationally intensive, requiring a large amount of time to analyse even six data points. Further, the intervals will often be quite large unless repeated samples are used which is not typically feasible in clinical settings. They also suffer from a similar problem to cosinor in that they always estimate the parameters, even when no oscillation is present. This can be mitigated by the researcher applying thresholds to the parameter estimates, but these are typically chosen subjectively.

Certain techniques from other fields for detecting oscillations have been adapted for biological data [119]. One such technique is Fourier analysis [126,127]; Fourier analysis decomposes the data to estimate which period, from a list of pre-specified periods, best fits the data. The decomposition technique means outliers have less influence on the period prediction. Further, approximate *P*-values can be calculated, increasing confidence in the period estimate and allowing this technique to report when no oscillation can be detected [127]. Additional techniques are required to estimate the amplitude and phase if an oscillation is detected often requiring smoothing filters which can be computationally intensive [128].

Some algorithms, e.g., MetaCycle [129], have tried to harness the advantages of each approach into a single analysis method. Such combination algorithms can intelligently prioritise which constituent technique performs best on different types of data (e.g., evenly sampled, missing observation, frequency of samples, etc.) to provide performance that is better than the sum of its parts [19]. This suggests that a two-stage approach that combines pre-existing methods could provide an effective method to analyse clinical circadian data [126].

## Hurdles and future directions

### Limitations of algorithms

The algorithms discussed above have strong performance in healthy participants, often in regulated conditions, with well-sampled data. However, when these algorithms have been applied to datasets of critically ill patients, conflicting results have been concluded [130–135]. This may be because current algorithms are not designed to handle a small number of data points and can be easily influenced by outliers. This leaves an open question regarding the best algorithm to interpret such data. Due to the abundance of novel algorithms that currently exist, a two-step approach that combines existing statistical techniques may yield superior results rather than requiring entirely new methodologies [126].

To minimise any adverse effects caused to critically ill patients by repeatedly drawing blood samples, alternative approaches have been explored where sampling occurs at only one or two time points. Recent developments in machine learning have produced algorithms such as ZeitZeiger [136], BodyTime [137], TimeSignature [138], and BIO_CYCLE [139] that can estimate the internal circadian time of patients using a single blood sample. These methods have been shown to be as accurate as the analysis of dim-light melatonin onset and estimate phase with 1-h accuracy. However, these machine learning approaches were trained and validated on healthy volunteers in a constant routine. Thus, their performance when analysing patients who are experiencing disruption may be unreliable. One study has tested their performance in the clinical setting, finding the accuracy to be suboptimal [134]. Consequently, such techniques need further validation in this setting. One approach may be to fuse traditional circadian techniques together. This has been done for ClinCirc where the Lomb–Scargle periodogram is combined with Cosinor analysis [122]. Benchmarking has revealed that ClinCirc has a greater accuracy compared with traditional techniques. Further, it was used to describe an association between dampening of circadian oscillations and inflammation, a feature widely reported in animal models.

Another alternative would be to use single-cell heterogeneity to identify both the degree of circadian disruption and possible circadian-regulated targets. There are already methods to estimate oscillatory characteristics at the single-cell level. However, these appear to struggle at predicting circadian parameters. Thus, novel approaches are required. One such approach is Tempo, which is based on a Bayesian inference approach and uses single-cell RNA-sequencing to infer phase and hence internal circadian time [140].

### Possible Interventions

As a multi-component mechanism regulating a variety of biological pathways, the circadian clock provides an exciting avenue for pharmacological intervention with potentially fewer off-target effects. Depending on the condition, drugs that target amplitude, phase or period could have real therapeutic potential [141]. As an example of this, compounds targeting REV-ERBα/β have been extensively studied in the lung due to the role this axis has in the pathogenesis of the respiratory diseases described above. The small molecule REV-ERB agonist GSK4112 has been shown to repress inflammation in alveolar macrophages *in vitro* [18,81] and collagen secretion in an *ex vivo* lung slice model of fibrosis [75]. Further, a related REV-ERB compound with improved bioavailability, SR9009 has shown potential *in vivo*, reducing cigarette smoke-induced inflammation in the lungs [58]. Compounds targeting ROR have also been shown to be effective in models of respiratory disease, which have been recently reviewed [142]. Therefore, targeting circadian biology is likely to have significant translational impact in the future.

## Conclusion

It is clear from studies described above that circadian regulation of respiratory biology is likely to affect several respiratory diseases. Although the mechanisms for these effects are well described in animal models, translation of these effects into patients has been hampered by a lack of robust methods to ascertain circadian disruption. This is likely to be translationally important as although we cannot control what time exposure to aetiological agents occurs the underlying mechanisms for these effects could be targeted through lifestyle changes or chemical compounds reducing disease severity and associated mortality. To prove the effectiveness of interventions it will also be useful to develop mathematical techniques or single time sampling methods so that the effect of interventions targeting the molecular oscillator can be accurately evaluated. Once we can quantify circadian disruption in patients, this will reveal translational effects [122] facilitating deployment of circadian logic and circadian compounds in clinical care.

## Data Availability

Not applicable since a review article.

## Open Access

Open access for this article was enabled by the participation of University of Manchester in an all-inclusive *Read & Publish* agreement with Portland Press and the Biochemical Society under a transformative agreement with JISC.

## Abbreviations

COPD: chronic obstructive pulmonary disease
ICS: inhaled corticosteroids
IPF: idiopathic pulmonary fibrosis
LPS: lipopolysaccharide
PCLS: precision cut lung slice
TTFL: transcription-translation feedback loop
